# Supplementary material for: Explainable Deep Hypergraph Learning Modeling the Peptide Secondary Structure Prediction
Source: Adv Sci (Weinh). 2023 Feb 15;10(11):2206151. doi: 10.1002/advs.202206151 (PMC10104664; doi:10.1002/advs.202206151)
Supplement: Supplementary file 1 — Supporting Information [file ADVS-10-2206151-s001.pdf]

ADVANCED SCIENCE
Open Access

Supporting Information

for *Adv. Sci.*, DOI 10.1002/advs.202206151

Explainable Deep Hypergraph Learning Modeling the Peptide Secondary Structure Prediction

Yi Jiang, Ruheng Wang, Jiuxin Feng, Junru Jin, Sirui Liang, Zhongshen Li, Yingying Yu, Anjun Ma, Ran Su, Quan Zou, Qin Ma and Leyi Wei**

Explainable deep graph learning accurately modeling the peptide secondary structure prediction

Yi Jiang[1,2#], Ruheng Wang[1,2#], Jiuxin Feng[1,2], Junru Jin[1,2], Sirui Liang[1,2], Zhongshen Li[1,2], Yingying Yu[1,2], Anjun Ma[3], Ran Su[4], Quan Zou[5], Qin Ma[3*] and Leyi Wei[1,2*]

[1]School of Software, Shandong University, Jinan, China

[2]Joint SDU-NTU Centre for Artificial Intelligence Research (C-FAIR), Shandong University, Jinan, China

[3]Department of Biomedical Informatics, College of Medicine, The Ohio State University, Columbus, OH, 43210, USA

[4]College of Intelligence and Computing, Tianjin University, Tianjin, China

[5]Institute of Fundamental and Frontier Sciences, University of Electronic Science and Technology of China, Chengdu, China

[#]These authors contributed equally to this work as first authors.

*Corresponding authors:

Q.M: qinma@osumc.edu

L.W: weileyi@sdu.edu.cn

Supplementary Materials

Supplementary Tables

Supplementary Table 1. Summary of the datasets for peptide secondary structures of three states.

Datasets	Structural states			Sequence number
	H	E	C	
Training set	33,455	16,901	29,177	1,028
Testing set	5,294	1,119	3,733	257

Supplementary Table 2. Summary of the datasets for peptide secondary structures of eight states.

Datasets	Structural states								Sequence number
	H	G	I	E	B	T	S	C	
Training set	48,132	7,262	35	49,205	2,994	24,006	22,394	46,940	1,018
Testing set	1,616	150	0	1,089	86	707	513	966	42

Note: We randomly selected 1,018 sequences out of all 1,060 sequences as the training set and the remaining 42 sequences as our testing set.

Supplementary Table 3. The experimental results of all the methods on independent test set

Method	$Observed_j$	Predicted			Acc_j (%)	Acc (%)	SOV (%)
		H	E	C			
Jpred	H	4,195	146	953	79.24	78.05	60.62
	E	64	467	588	52.54		
	C	337	259	3,136	84.03		
PSSP-MVIRT	H	4,773	99	422	90.16	78.50	75.81
	E	139	636	344	56.84		
	C	836	341	2,556	68.47		
PROTEUS2	H	4,656	81	557	87.95	82.45	72.61
	E	34	770	315	68.81		
	C	364	430	2,913	78.73		
RaptorX	H	4,493	142	659	84.87	82.98	78.39
	E	27	693	399	61.93		
	C	306	194	3,233	86.61		
PHAT	H	4,716	61	517	89.08	**84.07**	**79.78**
	E	49	803	267	**71.76**		
	C	482	240	3,011	80.66		

Supplementary Table 4. The experimental results of AlphaFold, trRosetta, and our method on independent testing set

Method	Acc(%)	Time cost(h)
AlphaFold	84.10	4.52
trRosetta	83.74	5.03
PHAT	**84.07**	**1.07**

Note: We input our testing set into AlphaFold and trRosetta and extract secondary structure from their predicted PDB files using DSSP algorithm. Besides, the time costs are recorded for better comparison.

Supplementary Table 5. Results of the models with different encoding strategies.

Method	$Observed_j$	Predicted			Acc_j (%)	Acc (%)	SOV (%)
		H	E	C			
HyperGMA	H	4,698	294	302	88.74	63.04	49.08
	E	191	192	736	65.77		
	C	1,256	1,515	962	25.77		
ProtT5	H	4,814	133	347	90.93	82.30	73.99
	E	68	873	178	78.02		
	C	629	440	2,664	71.36		
HyperGMA(+)ProtT5	H	4,726	73	495	89.27	82.71	74.14
	E	66	776	277	69.35		
	C	553	290	2,890	77.42		
TextCNN(+)ProtT5	H	4729	89	476	89.32	81.95	76.34
	E	64	800	255	71.49		
	C	606	341	2786	74.63		
TextCNN(*)ProtT5	H	4548	58	688	85.91	82.33	76.08
	E	66	679	374	60.68		
	C	442	165	3126	83.74		
HyperGMA(*)ProtT5	H	4,716	61	517	89.08	**84.07**	**79.78**
	E	49	803	267	71.76		
	C	482	240	3,011	80.66		

Note: (+) represents the fusion of encoding features with the element-wise multiplication strategy, and (*) represents the fusion of encoding features with the element-wise additive strategy.

Supplementary Table 6. Results of our model with different training strategies.

Method	Observed$_j$	Predicted			Acc_j	Acc	SOV
		H	E	C	(%)	(%)	(%)
Cross Entropy loss function	H	4,723	91	480	89.21	83.24	77.32
	E	63	801	255	71.58		
	C	537	274	2,922	78.27		
CRF score function	H	4,716	61	517	89.08	84.07	79.78
	E	49	803	267	71.76		
	C	482	240	3,011	80.66		

Supplementary Table 7. The results of comparison in prediction of peptide toxicity.

Method	SN	SP	FDR	FPR	Acc	MCC
ATSE (original method)	95.11% (+0.12%, -0.12%)	92.72% (+0.12%, -0.13%)	8.72% (+0.13%, -0.13%)	7.81% (+0.13%, -0.13%)	94.13% (+0.14%, -0.13%)	89.07% (+0.12%, -0.14%)
ATSE (PSSP-MVIRT)	94.81% (+0.42%, -0.40%)	93.03% (+0.42%, -0.44%)	8.62% (+0.40%, -0.43%)	7.99% (+0.41%, -0.41%)	93.86% (+0.41%, -0.41%)	87.75% (+0.40%, -0.44%)
ATSE (PROTEUS2)	94.89% (+0.20%, -0.21%)	93.22% (+0.18%, -0.20%)	7.90% (+0.19%. -0.19%)	7.24% (+0.21%, -0.20%)	94.31% (+0.20%, -0.20%)	89.12% (+0.19%, -0.18%)
ATSE (PHAT)	95.06% (+0.20%, -0.21%)	93.4% (+0.22%, -0.23%)	8.51% (+0.21%. -0.21%)	7.53% (+0.20%, -0.22%)	94.74% (+0.20%, -0.20%)	89.62% (+0.20%, -0.24%)

Note: We report the average after performing each experiment 20 times by splitting the data set for other methods based on the data set of ATSE.

Supplementary Table 8. The results of comparison in prediction of T-cell receptor interactions with MHC-peptide complexes.

Method	Acc	Precision	Recall	F1-score
NetTCR-2.0 (original method)	93.43% (+1.07%, -3.23%)	42.03% (+4.98%, -11.23%)	78.67% (+4.44%, -4.47%)	54.68% (+4.93, -10.78%)
NetTCR-2.0 (PROTEUS2)	93.45% (+2.25%, -4.05%)	43.25% (+11.55%, -15.75%)	79.21% (+3.39%, -5.01%)	55.47% (+8.33%, -12.27%)
NetTCR-2.0 (PSSP-MVIRT)	93.66% (+2.44%, -2.65%)	43.93% (+14.57, -9.83%)	78.83% (+3.7%, -6.7%)	56.01% (+10.69%, -10.71%)
NetTCR-2.0 (PHAT)	94.04% (+2.76%, -2.54%)	45.54% (+19.26, -10.44%)	78.6% (+6.57%,1.53%)	57.29% (+13.81%, -7.99%)

Note: We report the average after performing each experiment 20 times by splitting the data set for other methods based on the data set of NetTCR-2.0.

Supplementary Table 9. The results of comparison in prediction of protein-peptide binding sites.

Method	AUC	MCC
PepBCL (SPOT-1D-Single)	78.6%	35.7%
PepBCL (PROTEUS2)	79.02%	31.3%
PepBCL (PSSP-MVIRT)	78.7%	30.9%
PepBCL (PHAT)	79.6%	36.0%

Note: We report the average after performing each experiment 20 times by splitting the data set for other methods based on the data set of PepBCL.

Supplementary Table 10. The experimental results of PHAT and SSpro8 on independent test set for eight states.

Method	Acc_j(%)								Acc	SOV
	H	G	I	E	B	T	S	C	(%)	(%)
PHAT	78.06	67.78	0	77.55	63.21	76.10	68.32	74.37	75.49	76.11
SSpro8	79.52	66.74	0	79.19	62.45	69.47	70.18	76.33	75.19	73.62

Supplementary Table 11. Summary of the datasets for peptide distance/contact map

Datasets	Max length	Min length	Average length	Sequence number
Training set	100	31	73	2715
Testing set	99	30	72	200

Note: The peptide sequences are from SCRATCH-1D and the corresponding structures are extracted from Protein Data Bank.

Supplementary Figures

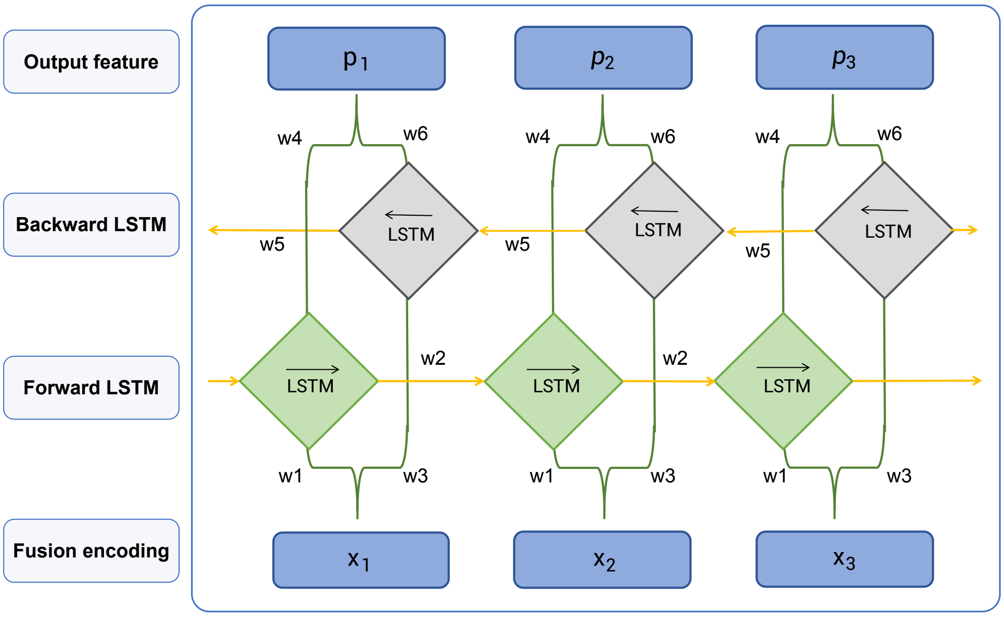

Supplementary Figure 1. Bi-LSTM Architecture. It can be shown that the forward layer and the backward layer are connected to the output layer, which contains shared weights w1-w6. In the forward layer, the forward calculation is performed from time 1 to time t, and the output of the forward hidden layer at each time is obtained and saved. In the backward layer, reverse the calculation from time t to time 1 to get and keep the output of the backward hidden layer at each time. Finally, the final output is obtained by combining the output results of the corresponding forward layer and backward layer at each time.

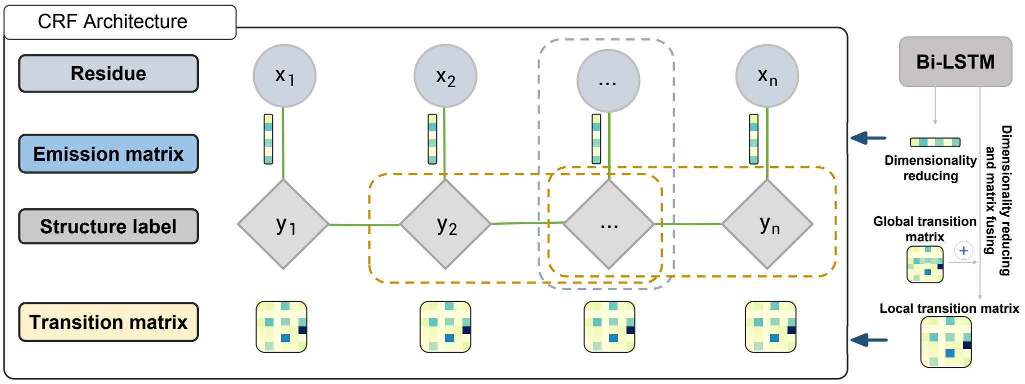

Supplementary Figure 2. CRF Architecture. The emission matrix consisting of the possibility of different sub-structures at each residue can be learned by Bi-LSTM layer. The local transition matrix is the fusion of the global transition matrix and the residue features from Bi-LSTM for transformation scoring among sub-structures.

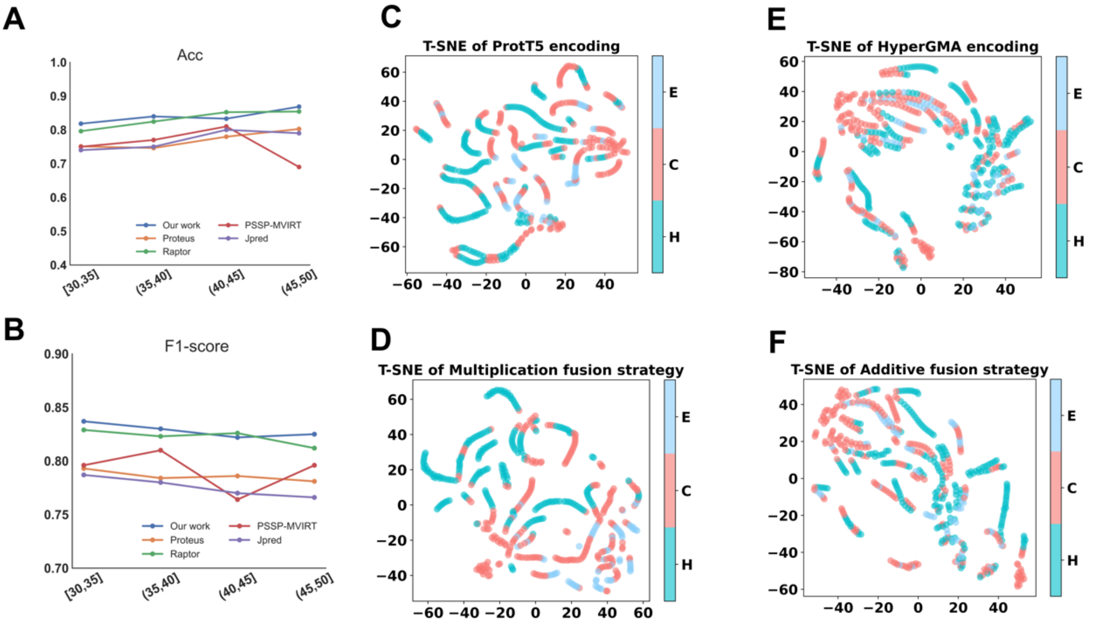

Supplementary Figure 3. (**A**) Acc is used as the evaluation metric; (**B**) F1-score is used as the evaluation metric. (**C–F**) represent t-SNE visualization results of the fused extractors in multiplication or additive and individual extractors of ProtT5, HyperGMA, respectively.

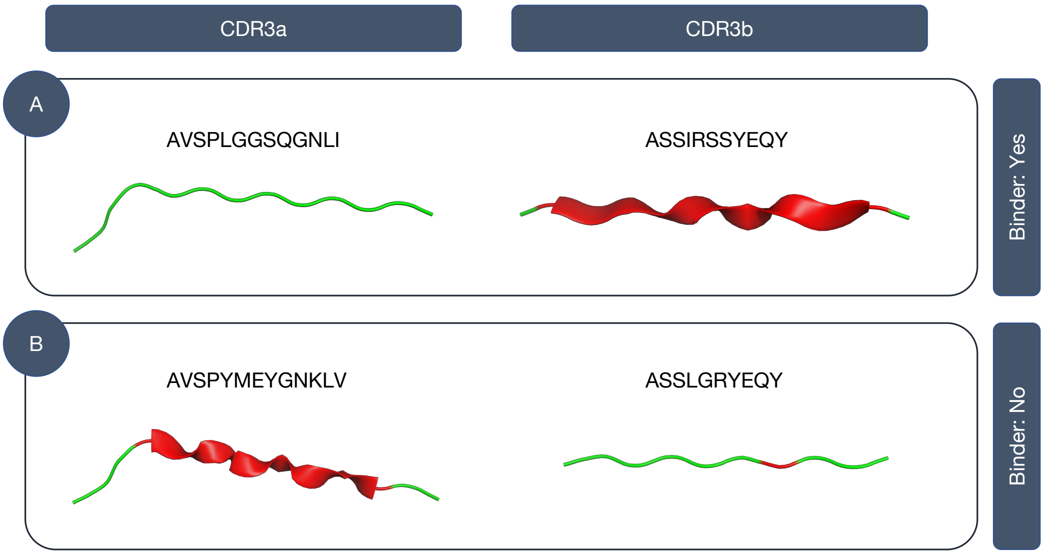

Supplementary Figure 4. Visualization of the secondary structures of the two peptide sequences predicted by our method.

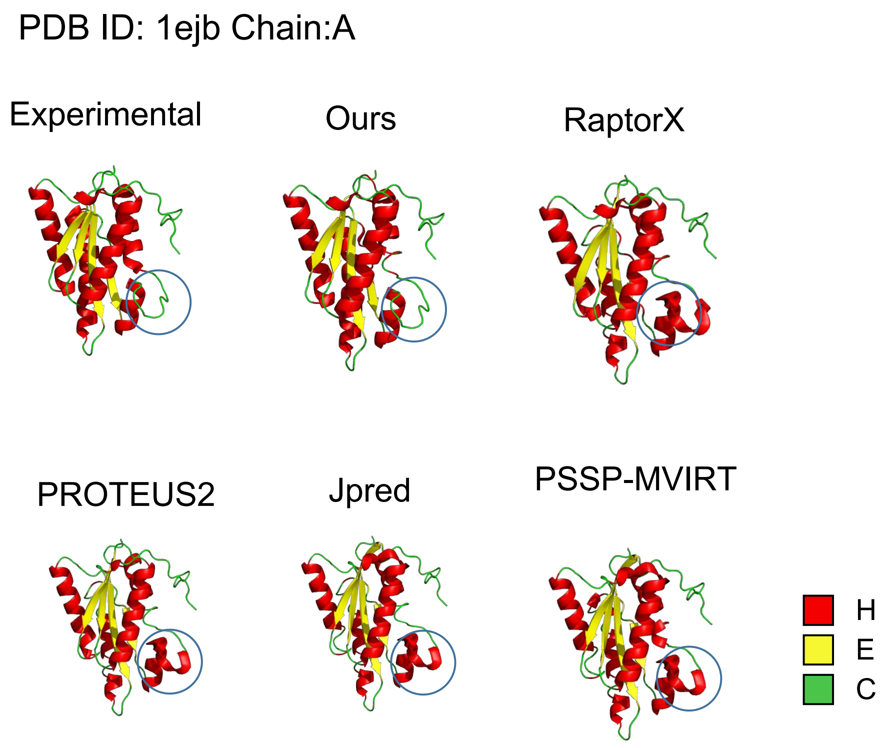

Supplementary Figure 5. Visualization of secondary structures mapped into tertiary structures for our method and existing methods including RaptorX, PSSP-MVIRT, PROTEUS2 and Jpred. The visualization of predictions by our method and existing methods for the peptide with PDB ID: 1ejb. The prediction regions with large differences from different methods are marked with circles.

Experimental settings

To train a robust and accurate model, we apply the layer normalization and dropout techniques. The layer normalization is used in integrating the features from the pretrained model ProtT5 and HyperGMA. Layer normalization can impose constraints on the "scale" problem, which may be caused by the embedding of multiple features in the learning process, effectively reducing the model variance. As for dropout, it is inserted into the attention layer of HyperGMA, solving the overfitting problem.

During slicing peptides into fragments and dividing fragments into residue groups to construct the structure of the hypergraph, we set 12 residues long as the length of the fragment, and there are four residues coincident between two neighboring fragments. As for the residues group, two residues are used to form a group with one same residue in the neighboring two groups. In our study, the whole deep learning models were trained globally by the Adam algorithm with a learning rate l = 1e-4 to minimize the cost function Loss. The training epoch is set to 200, and it performs best in the around 121 epoch. All the training and testing procedures were performed based on Nvidia RTX 3090 GPUs and implemented by python based on PyTorch.

Supplementary metrics

To evaluate the results of comparison in prediction of peptide toxicity, we used six traditional evaluation metrics commonly used in binary classification tasks, including Sensitivity (SN), Specificity (SP), False discovery rate (FDR), False positive rate (FPR), Accuracy (ACC) and Mathew's correlation coefficient (MCC). The metrics are calculated as follows:

$$SN = \frac{TP}{TP + FN} \tag{1}$$

$$SP = \frac{TN}{TN + FP} \tag{2}$$

$$FDR = \frac{FP}{TP + FP} \tag{3}$$

$$FPR = \frac{FP}{FP + TN} \tag{4}$$

$$ACC = \frac{TP + TN}{TP + TN + FP + FN} \tag{5}$$

$$MCC = \frac{TP \times TN - FP \times FN}{\sqrt{(TP + FN)(TP + FP)(TN + FP)(TN + FN)}} \tag{6}$$

where TP (true positive) and TN (true negative) represent the numbers of correctly predicted positive samples and negative samples, respectively; FP (false positive) and FN (false negative) represent the numbers of incorrectly predicted positive samples and negative samples, respectively. The metric SN measures the prediction ability of a predictor for positive samples, while the metric SP measures the ability of the predictor for negative samples. FDR calculates the proportion of errors in the positive samples predicted by the predictor, while FPR calculates the proportion of negative samples that are mistaken as positives by the predictor. ACC and MCC are used to evaluate the overall performance of a predictor. Moreover, the ROC (receiver operating characteristic) curve and PR (precision-recall) curve are often used to intuitively evaluate the overall predictive performance of a predictor. Here, we calculated the area under the ROC curve (AUC) to assess the overall predictive performance. The value of AUC is from 0.5 to 1. The larger the value of AUC, the better and more robust performance.

Data and code Availability

The authors declare that the data supporting the findings of this study are available within the article and its supplementary information files. For training details, we have listed the detailed experimental settings in **Experimental setting** and built a GitHub repository (https://github.com/jiangyi01/PHAT) to provide users with our source code and the tutorial, which includes details for the quick start of our method, the training environment, and downstream task pipelines, for using and reproducing our method easily. The benchmarking datasets are also available for downloading at http://inner.wei-group.net/PHAT/.
